# Association between adiponectin single nucleotide polymorphisms and the risk of diabetic polyneuropathy

**DOI:** 10.1038/s41598-025-86143-3

**Published:** 2025-01-31

**Authors:** Noha M. Bakr, Noha A. Hashim, Nevin F. Ibrahim, Sara F. Saadawy

**Affiliations:** 1 Biochemistry Department, Biotechnology Research Institute, National Research Centre (NRC), Dokki, Giza Egypt; 2 Neurology Department, Faculty of Medicine, Zagazig University, Zagazig, Egypt; 3 Internal Medicine Department, Faculty of Medicine, Zagazig University, Zagazig, Egypt; 4 Medical Biochemistry Department, Faculty of Medicine, Zagazig University, Zagazig, 44523 Egypt

**Keywords:** Biochemistry, Molecular biology, Neuroscience, Biomarkers, Neurology, Chemistry

## Abstract

Genetic factors play a significant role in the occurrence and clinical course of diabetic peripheral neuropathy (DPN). This research aimed to search the influence of adiponectin single nucleotide polymorphisms (SNPs) on the risk of developing and the severity of DPN in Egyptian patients. Adiponectin SNPs were genotype in 360 participants comprising diabetic sufferers with and without peripheral neuropathy and healthy volunteers via the polymerase chain reaction-restriction fragment length polymorphism (PCR-RFLP) approach. Regarding the + 45 T/G SNP, the TG/ and GG genotypes and the G allele were linked to an rised risk of DPN by comparing the DPN group with both the control and diabetic patients without peripheral neuropathy (DWPN) groups, and when comparing the DWPN group with the control group. Concerning + 276 G/T SNP, the GT genotype and T allele were linked to a declined risk of occuring DPN when comparing the DPN group with both other groups. Patients with DPN had greater frequencies of the GA genotype of the − 11,391 G/A SNP than individuals in the control group, while patients with DPN had greater frequencies of the AA genotype than patients in the DWPN group. Regarding clinic-pathological features, a meaningful rise in the mean values of fasting blood glucose (FBG), duration of the disease, and Toronto Clinical Neuropathy Severity Score (TCSS) were noted in the + 45 GG genotype and G allele carriers. Contrariwise, the + 276 TT genotype carriers had lower mean values for the same clinic-pathological features. For the T allele carriers, the same results were observed in case of duration of the disease and TCSS value. Our results concluded that adiponectin + 45 T/G SNP could be a risk factor considering DPN and the severity of the disease. The − 11391G/A SNP might be associated with DPN. In addition, + 276 G/T SNP could be a protective factor regarding DPN and the severity of the disease.

**Supplementary Information:**

The online version contains supplementary material available at 10.1038/s41598-025-86143-3.

## Introduction

Diabetes mellitus (DM) is the ninth prominent and principle reason of death in the past 30 years. It occurs due to either resistance to insulin action, insulin secretion, or both^1,2^. Globally, about 9.3% of adults currently have diabetes, and the prevalence will increase to 10.2% and 10.9% by 2030 and 2045^3,4^. In Egypt, T2DM affects roughly 15.56% of adults, with a corresponding yearly mortality rate of about 86,478, and it is expected that the prevalence of diabetes will be increased to 13.1 million by 2035^5^. DM is a complicated contact of systemic metabolic abnormalities like chronic hyperglycemia, local tissue reactions to toxic and harmful metabolites and dyslipidemia, which tends to trigger several macro- and microvascular complications leading to severe morbidities like nephropathy retinopathy, and neuropathy^6^.

Diabetic peripheral neuropathy (DPN) is among the greatest and widespread frequent, distressing, and earliest chronic microvascular complications of diabetes and affects various periphery nerve systems comprising motor and sensory neurons, causing considerable disability and poor quality of life^7,8^. It is often asymptomatic throughout its early stages; nevertheless, once symptoms and obvious deficits have been established, it cannot be reversed^9^. Consequently, early diagnosis and timely mediation are essential to stop its development and progression. Several factors comprising diabetes duration, hemoglobin A1C (HbA1C) level, smoking, and gender, along with genetic factors, might influence the onset and the course of the disease^7,10,11^. One of the attractive candidates related to a susceptibility region for T2DM, traits related to diabetes, metabolic syndrome, and cardiovascular disease is adiponectin^12–15^. Adiponectin is a protein hormone derived from adipocytes with a molecular weight of 30 kDa^12,16^. It may exert several vasculoprotective effects, including its special pleiotropic influences on endothelial cells, endothelial progenitor cells, smooth muscle cells and macrophages^17^. Moreover, it plays a critical role in protecting versus inflammation through the macrophages polarization directly towards anti-inflammatory phenotype, resulting in the healing of wound and the resolution of inflammatory events^18^. Besides, it modulates the inflammatory response of endothelial cells by decreasing tumor necrosis factor-α (TNF-α) production and stopping vascular remodeling via blocking smooth muscle cells proliferation and migration of^19^. In addition, it promotes the p38 mitogen-activated protein family of kinases (p38MAPK) and AMP-activated protein kinase (AMPK) and signaling pathways; thus, decreasing its plasma levels may interfere with the primary functioning of these essential pathways, inducing the development of diabetic phenotype^20,21^. Moreover, its low plasma levels changed lipid regulation causing Schwann cells collecting fat metabolites, which affects functions of peripheral nerve in diabetic sufferers^22^. Furthermore, abnormal levels of serum adiponectin are related to T2DM, obesity, cardiovascular diseases (CVD), insulin resistance, nephropathy, and neuropathy^23,24^. The adiponectin gene, which harbors three exons and two introns, is mapped on chromosome 3q27^25^. Numerous important single nucleotide polymorphisms (SNPs) in the gene of adiponectin are significantly correlated to the pathogenesis of diabetes^16,26^. The SNPs to be investigated were chosen according to the following criteria: proved SNPs for frequency in Genome Wide Association Studies (GWAS), and SNPs with scientific proof for their role in increased protein synthesis. Prior Studies have interested mostly on three SNPs, that were amongest the primary to be investigated via specialized resequencing attempts^27^. the gene’s immediate five flanking region contains − 11391G/ A (rs17300539), while exon 2 and intron 2 include + 45T/G (rs2241766), and + 276G/T (rs1501299), respectively^28^.

Although restricted prior studies investigated the linkage between the aforementioned adiponectin SNPs and the DPN risk^29–31^, the available inadequate documents and the unpredictable outcomes from these reports encouraged us toward considering a systematic and regular investigation of the adiponectin SNPs for their impact on the risk of DPN in T2DM patients. Thus, the current study aimed to obtain dependable approach to distinguish T2DM cases at high risk for DPN from others at low risk by determining the association of adiponectin SNPs, including + 45 T/G (rs2241766), + 276 G/T (rs1501299), and − 11,391 G/A (rs17300539) with the risk of DPN in our Egyptian patients. Moreover, the study examined SNPs’ prognostic significance in DPN sufferers by analyzing how they correlate with clinico-pathological features and disease severity.

## Subjects and methods

### Subjects design and description

The present case-control study involved 360 participants who were categorized into two groups: **Group I** consisted of 240 patients suffering from T2DM, which sub-grouped into 120 diabetic patients with peripheral neuropathy (DPN) and 120 diabetic patients without peripheral neuropathy (DWPN), and **Group II**included 120 apparently healthy individuals. Cases and healthy controls were recruited from the patients attending the outpatient diabetic clinic of Zagazig University Hospital, Internal Medicine Department, Faculty of Medicine, Zagazig University, Zagazig, Egypt from December 2021 to February 2023. The research ethical approval (reference number: ZU-IRB #9623/5-1-2022) was obtained by the Ethical Committee and the International Review Board (IRB), Faculty of Medicine, Zagazig University, and all procedures respected to the Declaration of Helsinki’s ethical rules. Written informed agree and consent was taken from all subjects shared in this study. The diagnosis of the disease was done followed the American Diabetes Association (ADA) criteria^32^ and aged between 30 and 50 years ago. The exclusion standards comprised the existence of autonomic or peripheral neuropathies from reasons other than diabetes, severe comorbidities (such as, recent cardiovascular events, heart failure, malignancies, and liver disease or advanced renal failure), and advanced peripheral arterial disease. In addition, patients receiving long-term immunosuppressive or immunomodulatory therapy, those with Type 1 diabetes mellitus (T1DM), those taking insulin, smokers, and those with hypertension were excluded from the research. Healthy subjects as a control group were randomly recruited from the general puplic at the same time. The collection of the complete personal information [such as age, gender, diabetes, and hypertension], a clinical examination [including comorbidity, blood pressure (BP), and blood glucose at the moment of neurological assessment], and anthropometric measurements [weight (kilograms), height (meters), and body mass index (BMI), waist circumference, and disease duration] were done by the direct contact with all contributors and from the outpatient medical files. Regular and routine clinical-laboratory data as fasting blood glucose (FBG), lipid profile, and hemoglobin A1C (HbA1c) were collected from medical record sheet.

### Diabetic polyneuropathy assessment

Diabetic peripheral neuropathy was evaluated by neurological examination and nerve conduction study (NCS) of upper and lower limbs and a combined clinical and electrophysiological score. NCS was reflected the abnormality if the abnormality value was ≥ 1 in two separate nerves^33^. Grouping of patients based on DPN was as follows: cases with no electrophysiological or clinical confirmation of neuropathy were characterized as normal, those with clinical signs or indications of neuropathy were characterized as probable clinical DPN, those with a mixture of clinical signs and indications of neuropathy were characterized as possible clinical DPN, those with abnormal NCS but without clinical signs nor indications of neuropathy were characterized as subclinical DPN, and those with a mixture of clinical signs and indications in addition to abnormal NCS was characterized as confirmed DPN.

The Toronto Clinical Scoring System (TCSS) was employed to monitor the severity DPN according to symptoms, reflexes, and sensory testing findings^34,35^. According to the TCSS score, the neuropathy was classified into 0–5 (indicating no neuropathy), 6–8 (indicating mild neuropathy), 9–11 (indicating moderate neuropathy), and 12 (indicating severe neuropathy).

## Methods

### SNP selection

These SNPs were chosen based on their established associations with adiponectin function and their potential relevance to diabetic complications including DPN according to previous studies^31,36–38^. The primer sequences and restriction enzymes for examining of each SNP were examined based on the free, on-line (https://blast.ncbi.nlm.nih.gov/Blast.cgi), and (https://nc2.neb.com/NEBcutter2/?noredir) version 2.0, respetively.

### Extraction of genomic DNA

By using the QIAamp DNA Blood Mini kit (Cat-No: #51104; Qiagen, Valencia, California, USA), DNA from the genome was isolated from EDTA- blood samples as defined by the manufacturer’s instructions. A NanoDrop spectrophotometer (ND1000; NanoDrop Technologies, Wilmington, Delaware, USA) and a 0.7% agarose gel electrophoresis were employed to assess the concentration and quality of DNA, respectively, and stored and frozen at −80^o^C until use.

### Genotyping of adiponectin + 45 T/G (rs2241766), + 276 G/T (rs1501299), and − 11,391 G/A (rs17300539) SNPs

All SNPs were genotyped using the polymerase chain reaction-restriction fragment length polymorphism (PCR-RFLP) method by using the following primers: for + 45 T/G (rs2241766), F: 5̀-GAAGTAGACTCTGCTGAGATGG-3̀ and R: 5̀-TATCAGTGTAGGAGGTCTGTGATG-3̀, for + 276 G/T (rs1501299), F: 5̀-TCTCTCCATGGCTGACAGTG-3̀ and R: 5̀-AGATGCAGCAAAGCCAAAGT-3̀ (38), and for − 11,391 G/A (rs17300539), F: 5̀-TTGGATGTCTTGTTGAAG-3̀ and R: 5̀TTTCGGATAACATTTTGACAGC-3̀ (36). SNPs’ PCR reactions were carried out in 20 µL of 10 µL Master Mix specific to PCR (Thermo Fisher Scientific, Massachusetts, USA), 1.5 µL of both primers (forward and reverse 10 pmol/µL), 4.5µL sterile deionized water, and 2.5µL of template DNA to produce the amplified PCR products.

To amplify PCR products, SNPs’ PCR reactions were conducted in 20 µL (1.5 µL of forward and reverse primers [10 pmol/µL], 10 µL Master Mix [Thermo Fisher Scientific, Massachusetts, USA], 4.5 µL deionised water, and 2.5 µL of template DNA) in a thermal cycler (Biometra, Gottingen, Germany) as follows: initial denaturation at 94^o^C for 5 min, followed by 40 cycles at 94^o^C for 30 s, (51^o^C for 30 s (for + 45T/G SNP), 52^o^C for 30 s (For + 276 G/T SNP) and 50^o^C for 30 s (for − 11391 G/A SNP)), and 72^o^C for 30 s, and a final extension of 7 min at 72^o^C. By using good quality FastDigest SmaI, BsmI, and MspI restriction enzymes(New England Biolabs, Massachusetts, USA) for 15 min at 37^o^C, the amplified PCR products were processed producing particulr fragments. In a gel documentation system (BioDocAnalyze, Biometra, Gottingen, Germany), the particular fragments were envisioned under UV light after being separated on a 1.5% agarose gel.

### Statistical analysis

The computional SPSS software (version 20; IBM SPSS Inc., Chicago, IL, USA) was employed for the data analysis. The sample size was calculated according to the statistical method by using the online, Web-based, free, open-source program OpenEpi calculator version 3.01 (OpenEpi, n.d.). At 80% study power and 95% CI to find differences at the α-level of 0.05, the estimated sample will be 120 subjects per group, by calculation of CramerV for calculation of effect size effect size = 0.65. Quantitative data outcomes were presented as mean ± standard deviation (SD). The outcomes of qualitative data were presented as numbers and percentages (N %). By applying the ANOVA test for quantitative variables, the variances in demographical data and laboratory parameters were compared between groups. The relationship between the genotype, allele, and the risk of diabetic polyneuropathy was examined via odds ratios (ORs) and their 95% confidence intervals (95% CIs). ANOVA (F test) was used for comparison the means of several groups. Tukey’s test was employed for comparison in between groups. Chi-squared test was employed to find the association between categorical variables. Logistic regression analysis was employed to find factors predicting the disease severity. The analysis of haplotype was applied via the Phase program^39^. The P value of 0.05 for all tests was reflected the significance.

## Results

### Demographical and clinico-laboratory data of studied groups

Table 1 includes the demographical and clinico-laboratory data of all studied groups. All three studied groups were matched regarding age and gender (*P* > 0.05). A significant difference was observed in all clinico-laboratory data when comparing diabetic patients with polyneuropathy with others (*P* < 0.001). Additionally, the same statistically significant difference was observed between the DWPN group and the control group (*P* < 0.001) apart from BMI and TG (*P* > 0.05). The mean duration of T2DM was highly significant in the DPN group when compared with the DWPN group (*P* < 0.001).

### Genotype and allele frequencies of adiponectin SNPs in all studied groups

#### Adiponectin SNPs in the DPN group and control group

**Fig. 1:**
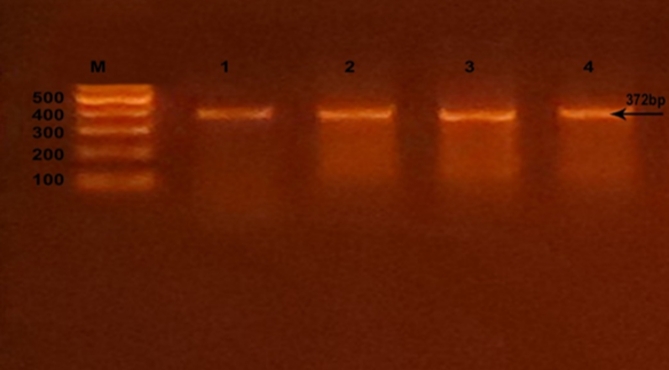
Agarose gel electrophoresis displaying the PCR amplification results of + 45 T/G (rs2241766) SNP in adiponectin gene. M: DNA marker (100 bp); Line 1–4: PCR products.

**Fig. 2:**
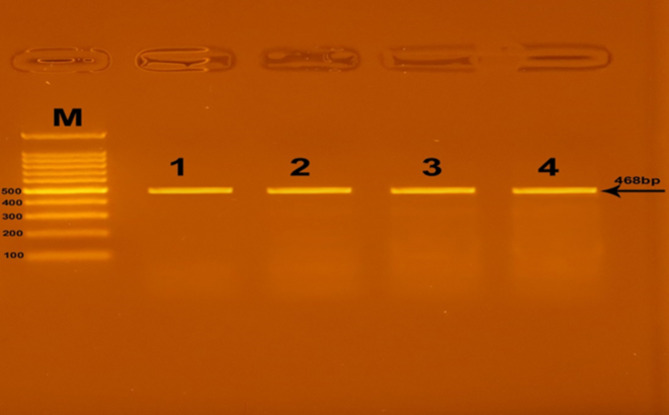
Agarose gel electrophoresis displaying The PCR amplification results of + 276 G/T (rs1501299) SNP in adiponectin gene. M: DNA marker (100 bp); Line 1–4: PCR products.

**Fig. 3:**
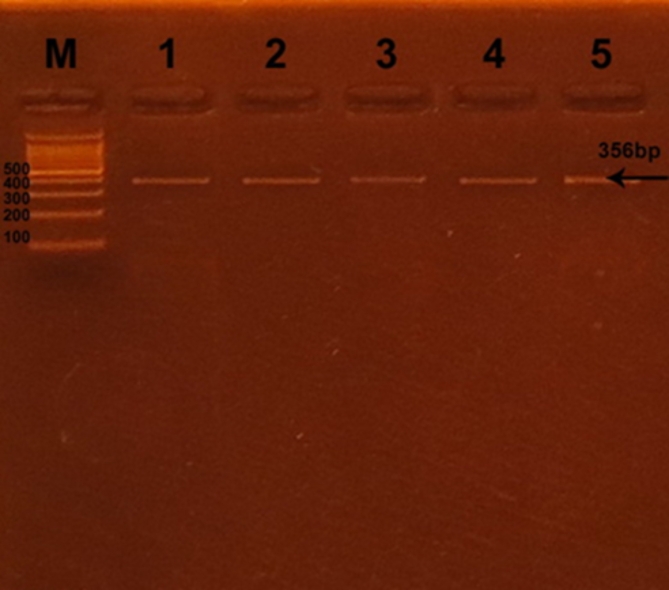
Agarose gel electrophoresis displaying the PCR amplification results of-11,391 G/A (rs17300539) SNP in adiponectin. M: DNA marker (100 bp); Line 1–5: PCR products.

**Fig. 4:**
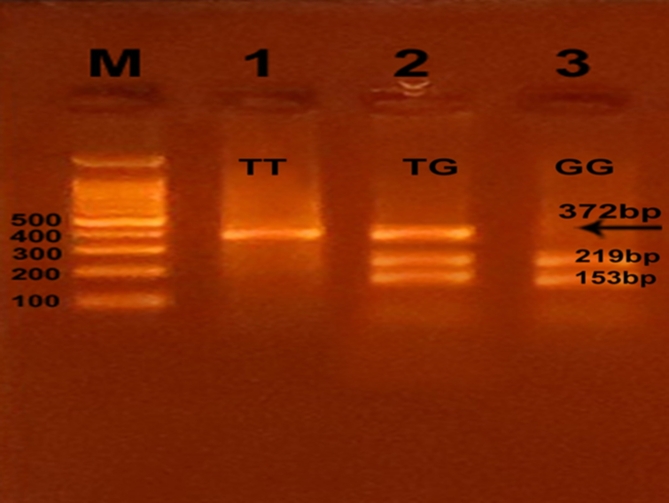
Agarose gel electrophoresis displaying the PCR-RFLP results for + 45 T/G (rs2241766) SNP in adiponectin gene. M: DNA marker (100 bp); Line 1: TT genotype; Line 2: TG genotype, and Line 3: GG genotype.

**Fig. 5:**
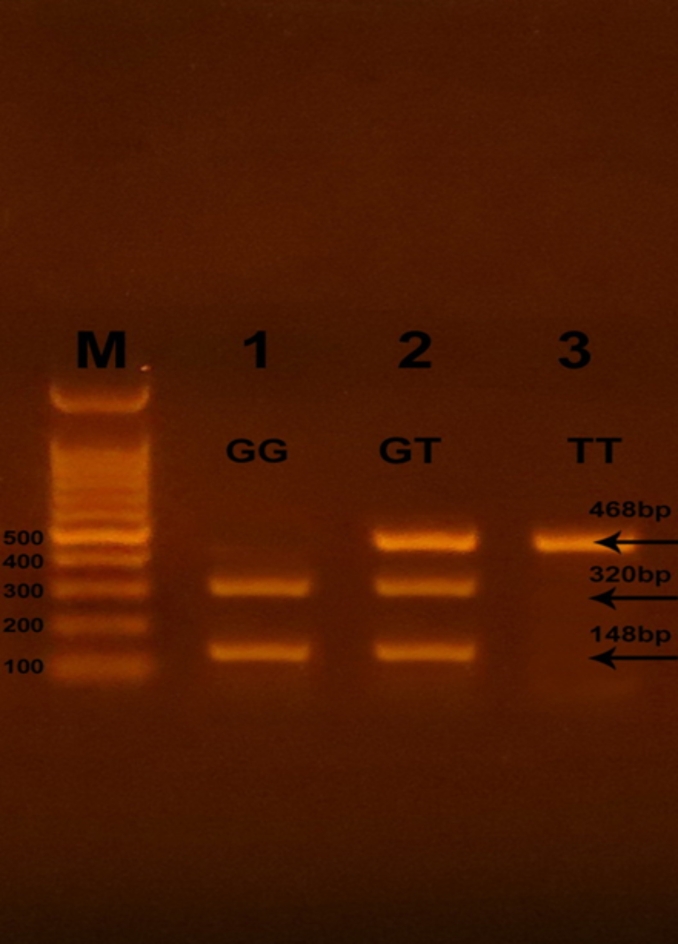
Agarose gel electrophoresis displaying the PCR-RFLP results for + 276 G/T (rs1501299) SNP in adiponectin gene. M: DNA marker (100 bp); Line 1: GG genotype, Line 2: GT genotype, and Line 3: TT genotype.

**Fig. 6:**
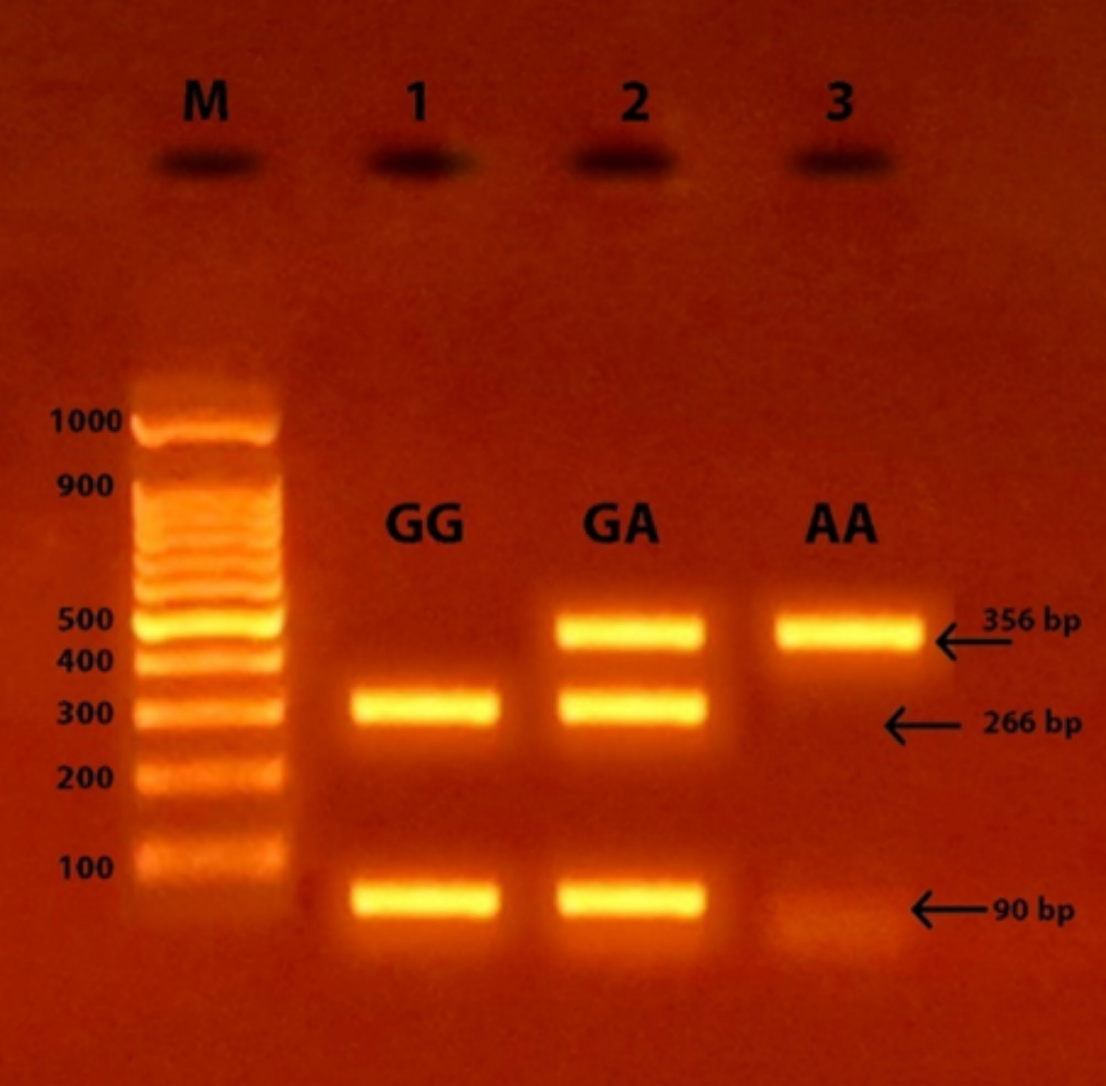
Agarose gel electrophoresis displaying the PCR-RFLP results for − 11,391 G/A (rs17300539) SNP in adiponectin gene. M: DNA marker 100 bp; Line 1: GG genotype, Line 2: GA genotype, and Line 3: AA genotype.

Successful amplification of the adiponectin + 45 T/G (rs2241766), + 276 G/T (rs1501299), and − 11391 G/A (rs17300539) SNPs was demonstrated by the presence of PCR amplified products with sizes of 372 bp (bp), 468 bp, and 356 bp, respectively (Figs. 1, 2, and 3). Regarding + 45 T/G (rs2241766) SNP, after digestion by restriction enzyme, the PCR-RFLP results were TT wild type homozygous (undigested product) (372 bp), TG heterozygous (372 bp, 219 bp, and 153 bp), and GG mutant homozygous (219 bp and 153 bp) (Fig. 4). In the case of + 276 G/T (rs1501299) SNP, GG wild type homozygous (digested fragment) (320 bp, 148 bp), GT heterozygous (468 bp, 320 bp, and 148 bp), and TT mutant homozygous (468 bp) were observed (Fig. 5). Concerning − 11391 G/A (rs17300539) SNP, GG genotype (266 bp, 90 bp), GA genotype (356 bp, 266 bp, and 90 bp), and AA genotype (356 bp) were detected (Fig. 6). According to Table 2, in the case of + 45 T/G SNP, the DPN group showed greater frequencies of TG and GG genotypes and G allele than in control group (56.7% vs. 31.7%, 31.7 vs. 15%, and 60% vs. 30.8%, respectively). In comparison to the TT genotype and T allele, the TG and GG genotypes and G allele were substantially linked to increased risk of developing DPN [odd ratios (ORs) (95% confidence intervals (CIs)^a^: 8.18 (2.76–25.1), P1 < 0.001; 9.65 (2.72–36.1), P1 < 0.001 and 3.36 (1.95–5.95), P1 < 0.001, respectively]. The GT genotype and T allele of + 276 G/T SNP were less frequent in the DPN group than in the control group (8.3% vs. 35% and 10.8% vs. 25.8%, respectively). Hence, the GT genotype and T allele were significantly linked to decreased risk of developing DPN as compared to the GG genotype and G allele [ORs (95% CIs)^a^: 0.16 (0.05–0.5), P1 < 0.001; 0.35 (0.16–0.74), P1 = −0.002, respectively]. The frequency of GA genotype and A allele of −11391 G/A SNP were higher in the DPN group when compared to the control group (55% vs. 36.7% and 44.2% vs. 30%, respectively). Hence, the GA genotype and A allele were considerably linked to a rised risk of occuring DPN as contrasted to the GG genotype and G allele [ORs (95% CIs)^a^: 2.74 (1.14–6.6), P1 = 0.012 and 1.85 (1.05–3.25), P1 = −0.02, respectively].

#### Adiponectin polymorphisms in DWPN and control group

The DWPN group had higher frequencies of + 45 GG genotype and G allele than the control group (31.7% vs. 15% and 46.7% vs. 30.8%, respectively). The GG genotype and G allele of + 45 T/G SNP were considerably related to a rising risk of occuring diabetes compared to the TT genotype and T allele [ORs (95% CIs)^b^: 2.94 (1.02–8.58), P2 = 0.02 and 1.96 (1.12–3.45), P2 = 0.01, respectively] (Table 3). When comparing the two groups, there are no significant differences in + 276 G/T and − 11,391 G/A SNPs.

### Adiponectin polymorphisms in DPN and DWPN

Regarding + 45 T/G SNP, the DPN group had significantly higher frequencies of TG and GG genotypes and G allele than the DWPN group. The TG and GG genotypes [ORs (95% CIs)^c^: 6.2 (2.02–19.7) and 3.29 (1.02–10.95), P3 < 0.001 and P3 = 0.02, respectively] and the G allele [OR (95% CI)^c^: 1.71 (1–2.96)] were linked to a statistically significant upregulated risk of DPN when compared to the TT genotype and T allele. Concerning + 276 G/T SNP, GT heterozygous and T allele were associated with protection from developing DPN when comparing both groups [ORs (95% CIs)^c^: 0.12 (0.04–0.3), P3 < 0.001 and 0.26 (0.13–0.55), P3 < 0.001, respectively]. Moreover, the AA genotype of −11,391 G/A SNP was more frequent in the DPN group than in the DWPN group, and this indicated that this genotype was associated with increased risk of DPN in our cohort (OR (95% CI)^c^: 4.51 (0.95–24.6), P3 = 0.03).

### Association between adiponectin SNPs and clinico-laboratory features in the DPN group

The numerous associations between the genotype frequencies of adiponectin gene SNPs and clinico-laboratory features in the DPN group are listed in Table 3. Regarding + 45 T/G SNP, at the genotypic level, a statistically significant increase in the mean values of FBG (*P* = 0.014), duration of the disease (*P* < 0.001), and TCSS [severity score] (*P* < 0.001) were noted in sufferes of GG genotype versus those of TT and TG genotypes. However, no significant relationship was found between different genotypes for other clinical data (*P* > 0.05). At the allelic level, a significant increase was observed in the disease duration (*P* = 0.04) and TCSS (*P* = 0.02) in G allele carriers when compared to T allele carriers. Inversely, the results revealed that patients with the mutant genotype (TT) had lower mean values for FBG (*P* = 0.04), disease duration (*P* = 0.013), and TCSS (*P* = 0.007) in the instance of the + 276 G/T SNP compared to those with the wild-type genotype (GG) and heterozygous genotype (GT). The same findings were observed at the allelic level, with a statistically significant decrease in the mean values of duration of the disease (*P* = 0.008) and TCSS (*P* = 0.002) in T allele carriers compared with G allele carriers. Regarding − 11,391 G/A SNP, only a statistically significant increase in the mean value of LDL cholesterol was observed in patients carrying the AA genotype (*P* < 0.001) and A allele (*P* < 0.001) compared to the GG genotype and G allele.

### Haplotype analysis of adiponectin + 45 T/G and + 276 G/T SNPs in the DPN and DWPN groups

Haplotype analysis was performed on the DPN and DWPN groups (Table 4). The results exhibited that the G^+45^-T^+276^ and T^+45^-T^+276^ haplotypes were significantly associated with a decreased risk of polyneuropathy (OR = 0.33, 95% CI 0.13–0.79; *P* = 0.006, and OR = 0.39, 95% CI 0.15–0.97; *P* = 0.02, respectively), while the G^+45^-G^+276^ haplotype was linked to the higher risk of polyneuropathy (OR = 4.3, 95% CI 1.74–11.1; *P* < 0.001).

### Haplotype analysis of adiponectin + 45 T/G and − 11,391 G/A SNPs in the DPN and DWPN groups

As presented in Table 5, the outcomes did not show any associations with polyneuropathy, except for the individuals carrying G^+45^-A^−11391^ haplotype was significantly linked to increased odd of polyneuropathy (OR = 2.1, 95% CI 0.95–4.67; *P* = 0.04).

### Haplotype analysis of adiponectin + 276 G/T and − 11,391 G/A SNPs in the DPN and DWPN groups

In Table 6, the findings revealed that the T^+276^-A^−11391^ and T^+276^-G^−11391^ haplotypes were significantly associated with a decreased risk of polyneuropathy (OR = 0.41, 95% CI 0.17–0.99; *P* = 0.029, and OR = 0.2, 95% CI 0.08–0.52; *P* < 0.001, respectively) compared to diseased groups.

### Multiple regression analysis for the disease severity prediction via TCSS

By applying multiple regression analysis, the disease duration, + 45 T/G, and − 11,391 G/A SNPs were recognized as predictors of increasing the severity of the disease according to their TCSS scores. Furthermore, + 276 G/T SNP was identified as a predictor of decreasing the severity of the disease as defined by their TCSS scores (Table 7).

## Discussion

Chronic hyperglycemia, inflammation, dyslipidemia, hypertension, and genetic factors play a significant role in developing DPN^40,41^. Several studies have focused on SNPs − 11,377 C/ G (rs266729) and − 11391G / A (rs17300539) in the promoter area, + 45 T / G (rs 2241766) in exon 2, and + 276 G /T (rs1501299) in intron 2. These four polymorphic variants are situated within the two linkage disequilibrium (LD) blocks of adiponectin: block 1, including the promoter sequence covering the area from − 14,811 to − 4120, and block 2, including the exons in the region − 450 to + 4545^37^. Thus, the adiponectin + 45 T/G, + 276 G/T, and − 11,391 G/A SNPs were selected in this study to obtain better insights about the possible link between these SNPs and the hazard of developing DPN in Egyptian patients. Despite the increasing proof that polyneuropathy symptoms are not a dependable sign of neuropathy in the progression of the disease, around 50% of cases are asymptomatic; therefore, they are susceptible to unconscious foot problems^39^. Thus, early recognition of individuals at high risk is extremely important. To address this demand, the current study has investigated the association between the abovementioned SNPs in the adiponectin gene and our population’s risk of developing DPN. To be aware, limited documents have investigated the association between the aforementioned adiponectin SNPs and the risk of DPN before, and the results were hazy and unclear, this guided us to carry out this work on such relations in our Egyptian cohort.

Considering + 45 T/G (rs2241766) SNP, the DPN group showed greater TG and GG genotypes and G allele frequencies than the control and DWPN groups. The DWPN group exhibited higher frequencies of GG genotype and G allele than the control group. Thus, our results indicated that individuals carrying TG and GG genotypes and G alleles could be more susceptible to developing DPN in our population. Reinforcing our findings, Ji et al.^29^observed that the frequency of the GG genotype in the DPN group was significantly higher than in the normal control group and non-diabetic peripheral neuropathy (NDPN) group. On the contrary, no significant association was observed when comparing the NDPN and control groups. Likewise our results, Mohammadzadeh and Zarghami^42^ and Motawi et al.^43^ in Iranian and Egyptian studies, respectively, stated that TG/GG genotypes and the G allele of + 45 SNP occured more repeatedly than the TT genotype and T allele in T2DM patients compared to the control group (*P* < 0.05). In addition, Li et al.^44^ reported that the wild type + 45 GG genotype was associated with the increased risk of T2DM in Japanese population. In contrast to our results, Jr. et al.^45^ stated that no suggestion for the association between the + 45T/G polymorphism and diabetic neuropathy, retinopathy, or nephropathy. Moreover, Hasani-Ranjbar et al.^29^ and Choe et al.^30^revealed that + 45 T/G SNP is not associated with diabetic neuropathy. One possible clarification of our findings is that this silent mutation, a synonymous mutation in exon 2 near the exon-intron junction, does not modify the amino acids sequence (GGT→GGG, Glycine→Glycine), and might impact adiponectin gene expression and circulating concentration of adiponectin via its linkage disequilibrium with another polymorphism in one of the introns that causes destabilization of the pre-mRNA, results in declined mRNA levels, and finally the development of pathophysiological effect and elevating phenotypic variability (DPN risk in T2DM patients)^37,46^.

Prior studies noted that the + 276 G/T SNP could play a protective role in T2DM, diabetic nephropathy and coronary artery disease (CAD) in different populations with T2DM^36,38,47^. In addition, Hara et al. 2002 also stated that the the wild type + 276GG genotype of the adiponectin gene was related to T2DM in Japanese study. Consistent with these results, our findings suggested that the heterozygote GT genotype and the mutant (T allele) were less frequent in the DPN group than in the control and DWPN groups. Hence, the GT genotype and T allele were significantly linked to the decreased risk of developing DPN and could confer protection against DPN in our Egyptian population. In disagreement with our results, Ji et al.^31^ observed that + 276 G/T (rs1501299) SNP could dramatically increase the chance of developing DPN in T2DM patients. Moreover, Choe et al.^30^showed that this SNP is not significantly linked to diabetic neuropathy prevalence. In agreement with our results, considering T2DM patients, several other literatures also did not note any association in the Chinese Han population^48–50^. By contrast, several studies observed that + 276 SNP was associated with T2DM in different populations^51–54^. The exact mechanism of how + 276 G/T SNP may be a protective factor in DPN is not completely well-defined. Several studies may clarify the match between our results and prior research on patients with diabetic nephropathy. Flyvbjerg^47^ and Demir et al.^55^indicated that diabetic microvascular complications are described through structural and functional organ damage according to vascular system modifications, which influence the capillaries and arterioles in the retina, kidney, and nerves. These changes include the wall thickening of small blood vessels, which causes bleeding and protein leakage, and the narrowing of blood vessels which reduces blood flow and impairs oxygen flow throughout the body, causing tissue or organ damage that are highly sensitive to oxygen levels like retina, kidney, and nerve cells. Moreover, the major destructive factor that is contributed to the structural and functional changes in the retina, kidneys, nerves, and vessels in people with diabetes is hyperglycemia. Hyperglycemia is an upregulation in the production of reactive oxygen species (ROS) with cytosolic NADPH oxidase and insufficiency in main antioxidants like reduced glutathione (GSH), which initially cause complications such as diabetic retinopathy (DR), diabetic nephropathy (DF), and diabetic neuropathy (DN)^56–59^. Genetically, the + 276 G/T SNP is an intronic mutation. However, there is a strong linkage disequilibrium and close association between this SNP and G90S missense mutation and other SNPs in the promoter region, respectively, representing that altering pre-mRNA splicing may cause downregulation of adiponectin expression^60^. In another words + 276 G/T is located distant from the common splice region inside intron 2 of the gene; with no well-known function. It may be an indicator of some other polymorphic modifications that influencing the gene expression. It has been observed that there was approximately complete linkage disequilibrium between this SNP and numerous genetic variations localized in the 3’ untranslated regions (3’UTR), which play a key function in the controlling of the gene expression via attaching proteins that control the processing, degradation or translation of mRNA^60^. Moreover, the mutant allele of this SNP (T allele) upregulated the plasma adiponectin levels and declined the insulin resistance^61^. Nevertheless, Kacso et al.^62^. observed that the TT genotype was associated with higher plasma adiponectin levels in T2DM patients. They speculated that although T2DM is characterized by “suppressed” adiponectin synthesis as reflected by lower adiponectin levels in T2DM patients in comparison to controls, diabetic carriers of the + 276 TT allele might be able to overcome this suppressed state in the presence of chronic inflammation that represents a characteristic of T2DM and a stimulus for adiponectin synthesis causing sufficiently increase in adiponectin levels. One interesting point is that diabetic patients carrying the T allele have better glycemic control than the other T2D subjects supporting a probable protective impact of this SNP. Moreover, another Spanish study reported that the adiponectin rs1501299 T allele upregulated plasma adiponectin levels and declined insulin resistance^61^.

Concerning − 11,391 G/A (rs17300539) SNP, the results showed that the frequencies of heterozygous GA genotype and mutated A allele were higher in the DPN group when compared to the control group. Moreover, the mutated AA genotype was more frequent in the DPN group than in the DWPN group, and this indicated that this SNP was associated with an increased risk of DPN development in our cohort. One study investigated the effect of this SNP on DPN risk in the Iranian population and reported the lack of association between the − 11,391 G/A SNP and diabetic microvascular complications, including neuropathy^29^. Our findings follow those reported by El-Shal et al.^38^ who reported that − 11,391 G/A SNP is associated with diabetic nephropathy in the Egyptian population. In addition, Hamza et al.^63^ revealed that − 1139 G/A SNP was associated with the diabetic nephropathy in Iraqi population from the middele Euphrates region. Regarding T2DM, Similar to our finding, Nomani et al.^64^ observed that − 11,391 G/ A SNP of adiponectin gene was not significantly linked to susceptibility to T2DM. Contrary to our outcomes, the conclusion of Vasseur et al.^65^ indicated that this SNP were significantly associated with T2DM in French Caucasian study. Moreover, Olckers et al.^66^noted that GA heterozygote in Black South African individuals with T2DM patients had a protective effect on the T2DM. Adiponectin − 11,391 G/A (rs17300539) polymorphism is situated in the intron 1. Although not portion of the promoter, it might be present on the sequence of enhancer. Sequences of enhancer could be situated in area of introns and as a result can modify the gene expression^67^. Furthermore, this SNP could provide otherwise spliced mRNA or influence the stability **of**mRNA^68^.

In this study, to examine the prognostic value of these SNPs in DPN patients, their associations with clinico-laboratory features in the DPN group were evaluated. Furthermore, our study is the first to focus on the effect of these SNPs on TCSS [severity score] to validate the possible role of adiponectin SNPs on the disease severity.

Considering the laboratory parameters, for + 45 T/G SNP, a statistically significant upregulate in the FBG mean values and duration of the disease were demonstrated in GG carriers and G allele carriers (except FBG) compared to TT and TG carriers and T allele carriers, respectively. In agreement with our results, Momin et al.^69^ revealed a higher level of FBG in TG and GG carriers than in the wild type. However, conflicting with our results, they observed a significant association between + 45 T/G SNP and higher BMI and TG in type 2 diabetics. Moreover, Hussain et al.^70^ and Joshaghani et al.^71^observed the association between the G allele in the + 45T/G SNP and the BMI increase. However, Ji et al.^31^, opposing our results, demonstrated no significant association between this SNP and the duration of diabetes and FBS in Korean T2DM with cardiovascular complications. Nevertheless, following our results, they demonstrated no significant differences between the + 45 T/G polymorphism and BMI, HbA1c, TG, and LDL. In addition, Palit et al.^14^ recorded the association of the + 45 T/G SNP with increased FBG in Gujarat diabetic patients.

Regarding + 276 G/T SNP, a statistically significant decline in the FBG mean values and duration of the disease were demonstrated in sufferers carrying the mutant (TT) genotype and the T allele (except FBG) in comparison to those with the wild type genotype (GG) and heterozygous genotype (GT) and G allele, respectively. Our observations are similar to the findings of Shramko et al.^72^who stated that + 276 GG genotype carriers had higher blood glucose levels than GT and TT carriers in T2DM patients. Inversely, another study found an association between this SNP with an increased mean value of FBG in Gujarat diabetic patients^14^. Moreover, Al-Nbaheen^73^ observed that this SNP in the adiponectin gene in the Saudi population with T2DM has no impact on any of the included baseline parameters like FBG, HbA1c, and lipid profile parameters.

Concerning − 113,919 G/A SNP, our results showed no significant association between any of the clinical features of DPN patients and this SNP. Only the LDL mean value revealed a statistically significant upregulation in the AA genotype and A allele carriers in comparison to the GG genotype and G allele carriers. Opposing to our results, Tabatabaei-Malazy et al.^74^noted a significantly higher BMI in women, and GA or AA carriers of − 11391G/A polymorphism in diabetic subjects when compared to non-diabetic subjects in the south-east of Iran. One explanation of our results is that the effect of SNPs of adiponectin on FBG (in case of + 45 T/G and + 276 G/T SNPs) and on LDL cholesterol (in case of −113919G/A SNP) may be attributed to the effect of adiponectin on glucose and lipid metabolism^70^. In addition, no association between these SNP and several of the above clinic-laboratory parameters related to DPN was observed. It is reasonable to infer that the genetic effects of these SNPs on T2DM are induced through changed adiponectin expression that affects some of the metabolic parameters^75^. The metabolic parameters might be detected by numerous genetic and environmental factors or lifestyle interferences in several populations and sample sizes^76^.

Considering the TCSS score and the disease severity, no prior study considered the relationship between the adiponectin SNPs and the disease severity in DPN patients. Our results revealed a statistically significant increase in the mean value of TCSS in + 45GG carriers and + 45G allele carriers compared to + 45TG and + 45TT carriers and + 45T allele carriers, respectively. Moreover, a statistically significant downregulate in the mean value of TCSS was observed in mutant genotype + 276TT and + 276T allele compared to + 276GT, + 276GG, and + 276G allele, respectively. These results revealed that the mutated genotype and + 45 T/G SNP allele are associated with increased disease severity. Besides, the mutated genotype and + 276 G/T SNP allele are associated with decreased disease severity in our population. Our data indicated the possible pivotal role of these adiponectin SNPs in the determination of disease severity in order to predict the progression of the disease. In addition, multiple regression analysis was performed to confirm our results and further analyze the relationship between the clinico-laboratory features of the disease and the studied SNPs and the TCSS in the DPN group to recognize the predictive factors that might account for the disease severity. It indicated that the duration of the disease and the + 45 T/G and − 11,391 G/A SNPs were identified as high-risk factors for the disease severity in DPN patients’ TCSS scores. Additionally, + 276 G/T is protective in the disease severity. Agreeing with our results, Szopa et al. [36] noted that the T allele of + 276 G/T was a protective risk factor, whereas the A allele of −11.391G/A was a risk factor concerning T2DM in Polish Caucasian patients.

Based on the haplotype analysis, our findings showed that G^+45^-T^+276^ and T^+45^-T^+276^ haplotypes were significantly associated with a decreased risk of polyneuropathy when comparing DPN and DWPN groups. Nevertheless, the G^+45^-G^+276^ haplotype was significantly linked to a higher risk of DPN when comparing the same groups. Similarly, Szopa et al.^36^ stated that the T^+45^-T^+276^ haplotypes were less frequent in the T2DM group than in the control group. Esteghamati et al.^77^ observed the same results in coronary artery disease in Iranian patients with type 2 diabetes. Moreover, Ji et al.^29^ found that G^+45^-T^+276^ haplotypes are negatively linked to the risk of DPN compared to the DPN and control groups. A recent study by Joshaghan et al. showed that the + 45G/+276G haplotype was linked to the increased risk of T2D in the Iranian population.

On the contrary, the same authors observed that the G^+45^-G^+276^haplotype was negatively associated with the risk of DPN when comparing the same groups. One explanation is that the + 45 T/G and + 276G/T SNPs are in a linkage disequilibrium block^28^. Furthermore, the two SNPs may be in linkage disequilibrium with other functional genetic loci that modify adiponectin production or its polymerization capability, altering its biological activity. In the case of haplotype analysis of adiponectin + 45 T/G and − 11,391 G/A SNPs in the DPN and DWPN groups, the individuals carrying G^+45^-A^−11391^haplotype were significantly associated with an increased risk of polyneuropathy. Our results may be attributed to the presence of strong linkage disequilibrium between this SNP and other SNPs of adiponectin gene like − 11,377 and − 11,391 SNPs in the 5′ promoter region that affects the expression, structure, or action of adiponectin^65^. Moreover, for haplotype analysis of adiponectin + 276 G/T and − 11,391 G/A SNPs in both groups, T^+276^-A^−11391^ and T^+276^-G^−11391^ haplotypes were significantly associated with a decreased risk of polyneuropathy.

Our current study is exceptional because no previous study has investigated the association between the adiponectin SNPs and the disease severity in DPN patients. Nevertheless, this study has a few potential limitations that should be taken into consideration. The first one, we carried out our study on Egyptian population, thus outcomes may not be appropriate to all populations and all ethnicities. Secondly, the study design was presented as a cross-sectional study, so we cannot give causality in the similar way as prior interventional literatures or longitudinal follow-up cohort studies. Therefore, our results cannot be concluded as complete information about adiponectin genetic polymorisms in all Egyptin population due to the small sample size. Finally, our study didn’t include the effect of environmental influences, lifestyle, and gene–gene interactions.

## Conclusion

In conclusion, our results showed that the adiponectin + 45 T/G SNP could be a risk factor for developing peripheral neuropathy in our Egyptian diabetic patients, impacting the disease severity considering TCSS score. The adiponectin − 11,391 SNP could be associated with DPN but not the severity of the disease. Moreover, adiponectin + 276 G/T SNP could be a protective factor regarding DPN risk and the severity of the disease. Nonetheless, In future work, we recommended that these SNPs should be recognized by structured prospective studies to analyze how these genetic mutations cause or accelerate DPN and their possible interaction with other risk factors. Thus, a definite assessment of the possible relationship probably needs the addition of more studies and larger sample sizes applied on DPN patients in different population and ethinities. Moreover, it is important to apply this genetic information to diabetic cases to determine the individuals at high risk for developing DPN to make precautionary strategies and modify the treatment protocol by selecting medication, dosage, and route of administration. Finally, future studies should be applied updated molecular technology techiques rather than PCR-RFLP to analyze the aformetioned SNPs.

**Table 1:** Demographical and clinico-laboratory data of studied groups.

	T2DM (*N* = 240)		Control group (*N* = 120)	
	DPN group (*N* = 120)	DWPN group (*N* = 120)		*P*-value
Demographical Data				
Age^a^	43.9 ± 7.6	41.6 ± 7.4	41.4 ± 6.6	0.12
Gender^b^ M/F	56 (47)/ 64(53)	56 (47)/ 64 (53)	60 (50)/60 (50)	0.91
Clinico-laboratory Data				
BMI (Kg/ m^2^)^a^	29.6 ± 2.15*	23.6 ± 3.7	24.1 ± 3.9	<0.001
FBG (mg/dl)^a^	238.5 ± 39.9*	130.5 ± 41.9^+^	104.5 ± 11.2	<0.001
LDL cholesterol ^a^	175.5 ± 14.5*	123.2 ± 19.1^+^	91.9 ± 8.8	<0.001
HDL cholesterol ^a^	37.8 ± 2.9*	47.4 ± 5.2^+^	48.2 ± 5.9	<0.001
TG^a^	239.7 ± 40.3*	191.1 ± 22.2	184.4 ± 17.6	<0.001
HBAIC^a^	9.3 ± 1.7*	7.5 ± 0.5^+^	4.7 ± 0.7	<0.001
Duration of disease ^a^	9.3 ± 3.6*	4.2 ± 1.7		<0.001
TCSS^a^	10.4 ± 3.2			

DPN, Diabetic peripheral neuropathy; DWPN, Diabetic without peripheral neuropathy; BMI, Body mass index; FBG, Fasting blood glucose; LDL, Low density lipoprotein; HDL, High density lipoprotein; TG, Triglyceride; HBAIC, Hemoglobin A1C; TCSS, Toronto Clinical Neuropathy Score. ^a^Data were reported as mean ± SD. ^b^ Data were reported as number (%). *P < 0.001 when comparing diabtic patients with peripheral neuropathy with others. ^+^*P* < 0.001 when comparing diabetic patients without peripheral neuropathy with control group.

**Table 2:** Genotype and allele frequencies of adiponectin SNPs in all studied groups.

SNP	DPN group (*N* = 120) *N* (%)	Control group (*N* = 120) *N* (%)	OR (95% CI)^a^	P1	DWPN group (*N* = 120) *N* (%)	OR (95% CI)^b^	P2	OR (95% CI)^c^	P3
**+ 45 T/G**									
**Genotypes**									
TT	14 (11.7)	64(53.3)	1.0 (ref.)		46 (38.3)	1.0 (ref.)		1.0 (ref.)	
TG	68 (56.7)	38 (31.7)	8.18 (2.76–25.1)	< 0.001**	36 (30)	1.32 (0.52–3.32)	0.51	6.2 (2.02–19.7)	< 0.001**
GG	38 (31.7)	18 (15)	9.65 (2.72–36.1)	< 0.001**	38 (31.7)	2.94 (1.02–8.58)	0.02*	3.29 (1.02–10.92)	0.02*
**Alleles**									
T	96 (40.0)	166 (69.2)	1.0 (ref.)		128 (53.3)	1.0 (ref.)			
G	144 (60.0)	74 (30.8)	3.36 (1.95–5.95)	< 0.001**	112 (46.7)	1.96 (1.12–3.45)	0.01*	1.71 (1- 2.96)	0.03*
**+ 276 G/T**									
**Genotypes**									
GG	102 (85)	68 (56.7)	1.0 (ref.)		58 (48.3)	1.0 (ref.)		1.0 (ref.)	
GT	10 (8.3)	42 (35)	0.16 (0.05–0.5)	< 0.001**	48 (40)	1.34 (0.5–3.1)	0.45	0.12 (0.04–0.3)	< 0.001**
TT	8 (6.7)	10 (8.3)	0.53 (0.11–2.5)	0.36	14 (11.7)	1.64 (0.41–6.8)	0.43	0.32 (0.07–1.39)	0.15
**Alleles**									
G	214 (89.2)	178 (74.2)	1.0 (ref.)		164 (68.3)	1.0 (ref.)		1.0 (ref.)	
T	26 (10.8)	62 (25.8)	0.35 (0.16–0.74)	0.002*	76 (31.7)	1.33 (0.73–2.42)	0.3	0.26 (0.13–0.55)	< 0.001**
**−11,391**									
**Genotypes**									
GG	34 (28.3)	62 (51.7)	1.0 (ref.)		46 (38.3)	1.0 (ref.)		1.0 (ref.)	
GA	66 (55)	44 (36.7)	2.74 (1.14–6.6)	0.012*	68(56.7)	2.08 (0.91–4.8)	0.057	1.32 (0.55–3.12)	0.49
AA	20 (16.7)	14 (11.7)	2.61 (0.74–9.43)	0.09	6 (5)	0.58 (0.1–2.09)	0.45	4.51 (0.92–24.6)	0.03*
**Alleles**									
G	134 (55.8)	168 (70.0)	1.0 (ref.)		160 (66.7)	1.0 (ref.)			
A	106 (44.2)	72 (30.0)	1.85 (1.05–3.25)	0.02*	80 (33.3)	1.17 (0.65–2.09)	0.57	1.58 (0.94–2.76)	0.085

OR (95% CI), Odd ratio and confidence intervals. P1 and OR (95% CI)^a^, Comparison between DPN and control groups; P2 and OR (95% CI)^b^: Comparison between DWPN and control groups, P3 and OR (95% CI)^c^: Comparison between DPN and DWPN groups.* *P* < 0.05 is considered significant. *P* > 0.05 is considered non-significant.

**Table 3:** Association of adiponectin SNPs and clinico-laboratory features in DPN group.

Genotype					Allele		
+ 45 T/G SNP	TT (*N* = 14)	TG (*N* = 68)	GG (*N* = 38)	*P*- value	T (*N* = 96)	G (*N* = 144)	*P*- value
**Clinico-laboratory Data**							0.33
BMI (kg/m2)	29.3 ± 2.3	29.6 ± 2.2	29.6 ± 2.1	0.93	29.2 ± 2.3	29.6 ± 2.2	0.17
FBG (mg/dl)	224 ± 30.7	241.5 ± 40	262.4 ± 29	0.014*	245 ± 39	235.3 ± 40	0.67
LDL cholesterol	182.7 ± 7	173.8 ± 16	175 ± 12.5	0.34	175.5 ± 15.6	174.3 ± 14.9	0.72
HDL cholesterol	38.7 ± 1.6	37.7 ± 3.3	37.6 ± 3.3	0.64	37.9 ± 3.1	37.7 ± 3	0.54
TG	252.8 ± 28.4	240 ± 40	233.9 ± 43	0.57	242.5 ± 38	238 ± 41	0.57
HBA1C	9.4 ± 1.6	9.2 ± 1.9	9.4 ± 1.5	0.9	9.2 ± 1.8	9.38 ± 1.7	0.04*
Duration of disease	7.8 ± 3.2	9.1 ± 3.0	12.1 ± 2.9**	< 0.001**	8.0 ± 3.1	9.3 ± 3.7	0.02*
TCSS	9.2 ± 1.9	9.8 ± 3.0	12.9 ± 2.4**	< 0.001**	9.2 ± 2.8	10.5 ± 3.3	

+ 276 G/T SNP	GG (*N* = 102)	GT (*N* = 10)	TT (*N* = 8)		G (*N* = 214)	T (*N* = 26)	
BMI (kg/m2)	29.4 ± 2.2	31 ± 1.8	29.8 ± 1.8	0.2	29.6 ± 2.2	30.6 ± 1.89	0.1
FBG (mg/dl)	271 ± 34.4	266.6 ± 49	233.1 ± 37.7	0.04*	249.5 ± 39	269 ± 40	0.09
LDL cholesterol	175.9 ± 12.6	172.6 ± 31	169.2 ± 9.2	0.62	175.2 ± 10.8	171.8 ± 15.4	0.31
HDL cholesterol	37.7 ± 2	38.3 ± 4	38.9 ± 3.3	0.89	37.6 ± 2.8	38.1 ± 3.0	0.54
TG	239.9 ± 41	219 ± 29	263 ± 34	0.26	238.3 ± 40	231 ± 39	0.53
HBA1C	9.2 ± 1.8	9.8 ± 2	9.0 ± 1.1	0.75	9.3 ± 1.7	9.7 ± 1.9	0.43
Duration of disease	9 ± 3.6*	6 ± 0.7	6.2 ± 0.9	0.013*	9.5 ± 3.6	6.7 ± 3.4	0.008*
TCSS	10.9 ± 3.2	7.8 ± 1.1	7 ± 0.0	0.007*	10.7 ± 3.3	7.8 ± 2.2	0.002

−11,391 G/A SNP	GG (*N* = 34)	GA (*N* = 66)	AA (*N* = 20)		G (*N* = 134)	A (*N* = 106)	
BMI (kg/m2)	29.7 ± 2	29.4 ± 2.4	29.6 ± 1.9	0.94	29.5 ± 2.2	29.6 ± 1.8	0.78
FBG (mg/dl)	226 ± 32	242.1 ± 43	247.4 ± 39	0.3	234.1 ± 38	244.5 ± 39	0.14
LDL cholesterol	160.4 ± 21*	175.7 ± 11.4	183.8 ± 8	< 0.001**	170.8 ± 15.4	178.2 ± 10	*P* < 0.001*
HDL cholesterol	37 ± 2.1	37.9 ± 3	38.8 ± 3.7	0.28	37.6 ± 2.8	38.1 ± 3.2	0.36
TG	237.4 ± 47	244 ± 40	227 ± 24	0.46	242.3 ± 42	240 ± 39	0.75
HBA1C	8.7 ± 1.6	9.7 ± 1.7	9.15 ± 1.9	0.14	9.3 ± 1.7	9.5 ± 1.7	0.52
Duration of disease	10.2 ± 2.7	9.1 ± 3.8	8.6 ± 4.4	0.44	9.4 ± 3.5	8.7 ± 3.9	0.3
TCSS	11.5 ± 2.8	10.3 ± 2	8 ± 2.2	0.1	10.7 ± 3.3	9.8 ± 3.4	0.14

Abbreviations: BMI, Body mass index; FBG, Fasting blood glucose; LDL, Low density lipoprotein; HDL, High density lipoprotein; TG, Triglyceride; HBAIC, Hemoglobin A1C; TCSS, Toronto Clinical Neuropathy Score. Data are expressed as mean ± SD. *P < 0.05 is considered significant. *P* > 0.05 is considered non- significant.

**Table 4:** Haplotype analysis of adiponectin + 45 T/G and + 276 G/T SNPs in DPN and NDPN groups.

Haplotypes	DPN group %	DWPN group %	OR (95% CI)	*P*- value
**G** ^**+45**^ **-T** ^**+276**^	20	43.3	0.33 (0.13–0.79)	0.006*
**G** ^**+45**^ **-G** ^**+276**^	83.3	53.3	4.3 (1.74–11.1)	< 0.001**
**T** ^**+45**^ **-T** ^**+276**^	18.3	36.7	0.39 (0.15–0.97)	0.02*
**T** ^**+45**^ **-G** ^**+276**^	61.7	61.7	**-**	**-**

**Table 5:** Haplotype analysis of adiponectin + 45 T/G and − 11,391 G/A SNPs in DPN and DWPN groups.

Haplotypes	DPN group %	DWPN group %	OR (95% CI)	*P*- value
**G** ^**+45**^ **-A** ^**−11391**^	61.7	43.3	2.1 (0.95–4.67)	0.04*
**G** ^**+45**^ **-G** ^**−11391**^	71.7	60	1.69 (0.74–3.89)	**0.17**
**T** ^**+45**^ **-A** ^**−11391**^	50	45	1.22 (0.56–2.67)	**0.58**
**T** ^**+45**^ **-G** ^**−11391**^	53.3	68.3	0.53 (0.23–1.19)	**0.09**

**Table 6:** Haplotype analysis of adiponectin + 276 G/T and − 11,391 G/A SNPs in DPN and NDPN groups.

Haplotypes	DPN group %	DWPN group %	OR (95% CI)	*P*- value
**T** ^**+276**^ **-A** ^**−11391**^	21.7	40	0.41 (0.17–0.99)	0.029*
**T** ^**+276**^ **-G** ^**−11391**^	15	46.7	0.2 (0.08–0.52)	< 0.001**
**G** ^**+276**^ **-A** ^**−11391**^	58.3	51.7	1.31 (0.6–2.87)	**0.46**
**G** ^**+276**^ **-G** ^**−11391**^	80	83.3	0.8 (0.29–2.22)	**0.63**

**Table 7:** Multiple regression analysis for prediction of disease severity by TCSS.

Variables	B	SE	*P*
**Disease duration**	0.59	0.08	< 0.001**
**+ 45 T/G**	1.14	0.08	< 0.001**
**+ 276 G/T**	−1.43	0.48	< 0.001**
**−11,391 G/A**	0.97	0.47	0.02*

B, Estimated unstandardized Beta regression coefficient; SE, Standard error. *P* < 0.05 is considered significant. *P* > 0.05 is considered non-significant.

## Electronic supplementary material

Below is the link to the electronic supplementary material.

Supplementary Material 1

Supplementary Material 2

## Data Availability

The datasets used and/or analysed during the current study data are provided within supplementary information files.

## Abbreviations

BMI: Body mass index
DF: Diabetic nephropathy
DM: Diabetes mellitus
DPN: Diabetic peripheral neuropathy
DR: Diabetic retinopathy
DWPN: Diabetic patients without peripheral neuropathy
HBA1C: Hemoglobin A1C
HDL: High-density lipoprotein cholesterol
LDL: Low-density lipoprotein cholesterol
NDPN: Non-diabetic peripheral neuropathy
PCR-RFLP: Polymerase chain reaction-restriction fragment length polymorphism
SNPs: Single nucleotide polymorphisms
T1DM: Type 1 diabetes mellitus
T2DM: Type 2 Diabetes mellitus
TCSS: The Toronto Clinical Scoring System
TG: Triglycerides
TNF-α: Tumor necrosis factor-α
