# Supplementary material for: Association between adiponectin single nucleotide polymorphisms and the risk of diabetic polyneuropathy
Source: Sci Rep. 2025 Jan 31;15:3872. doi: 10.1038/s41598-025-86143-3 (PMC11785776; doi:10.1038/s41598-025-86143-3)

16-Adipo+ 45 T-G After digestion

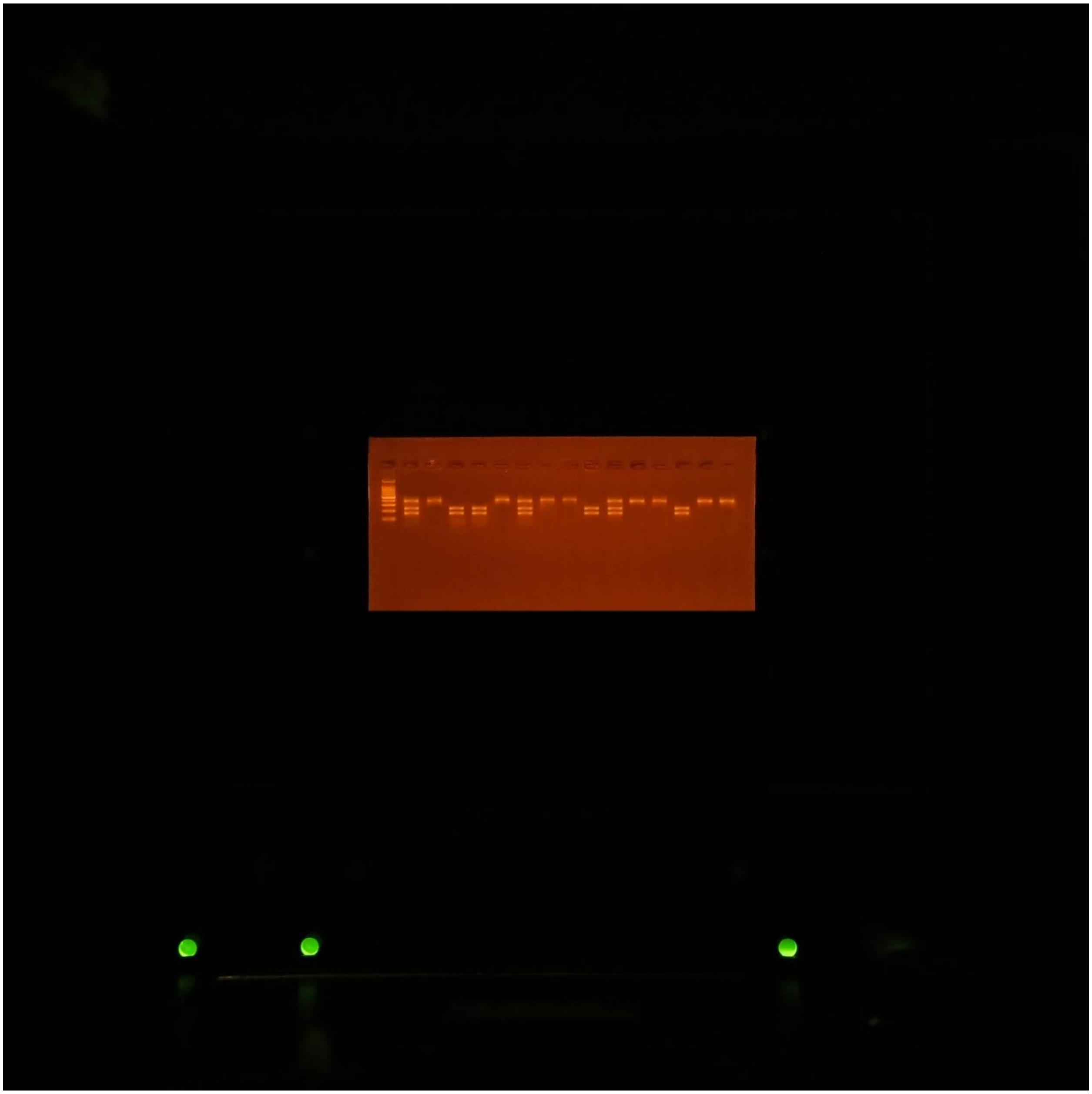

Adipo 267 PCR produc G-Tt

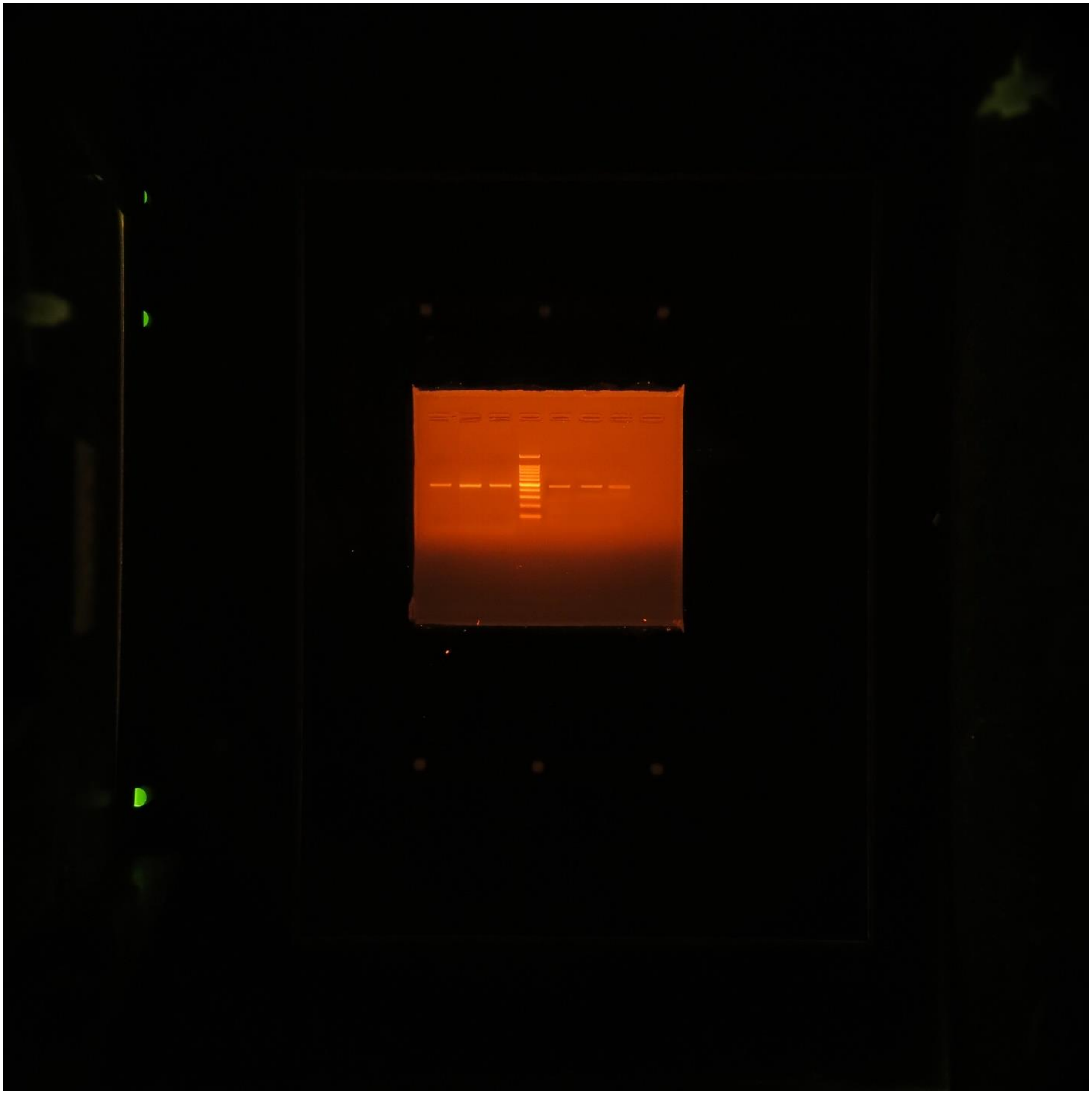

Adipo -11391 G-T PCR product

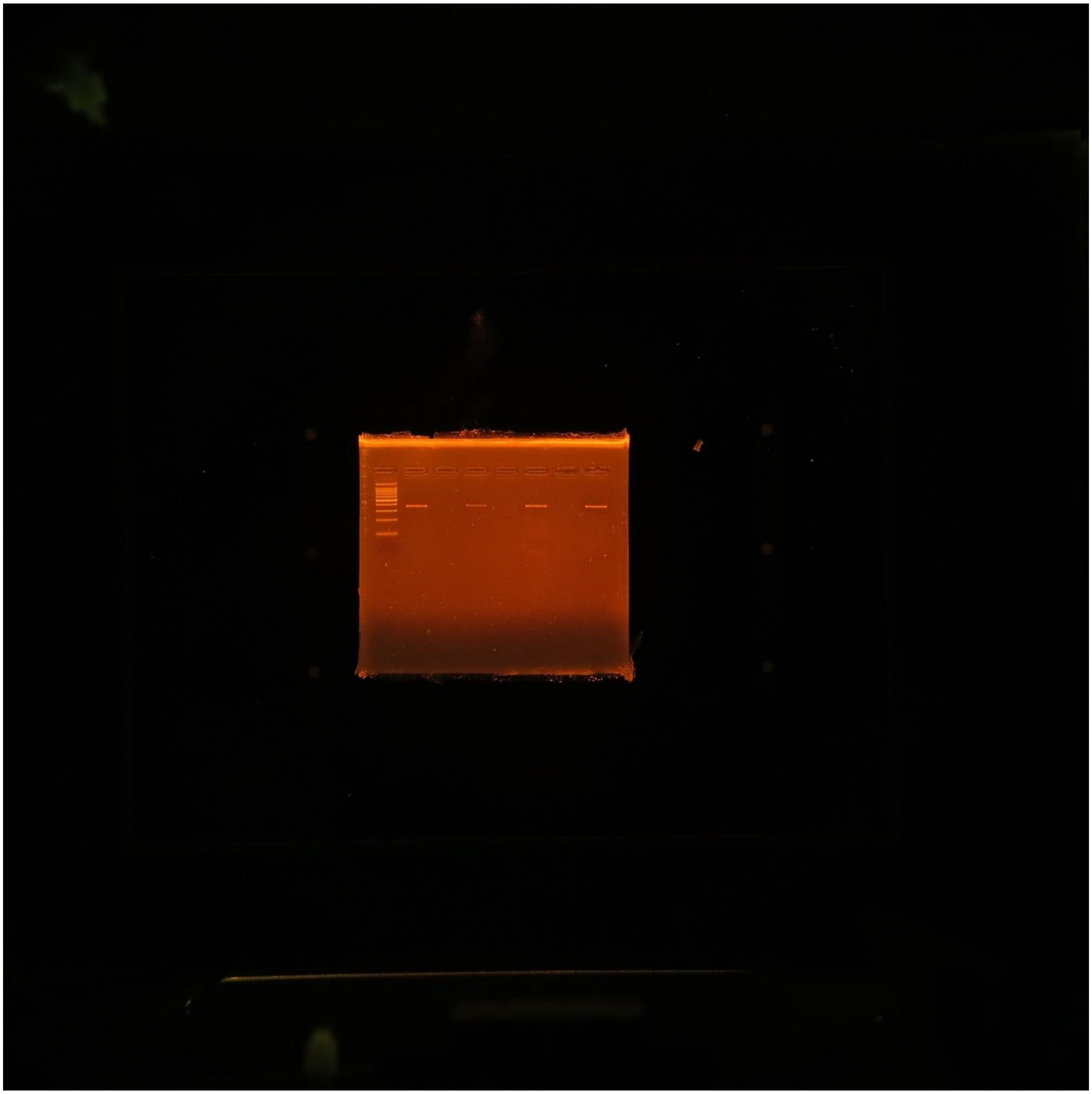

Adipo+ 267 G-A after digestion

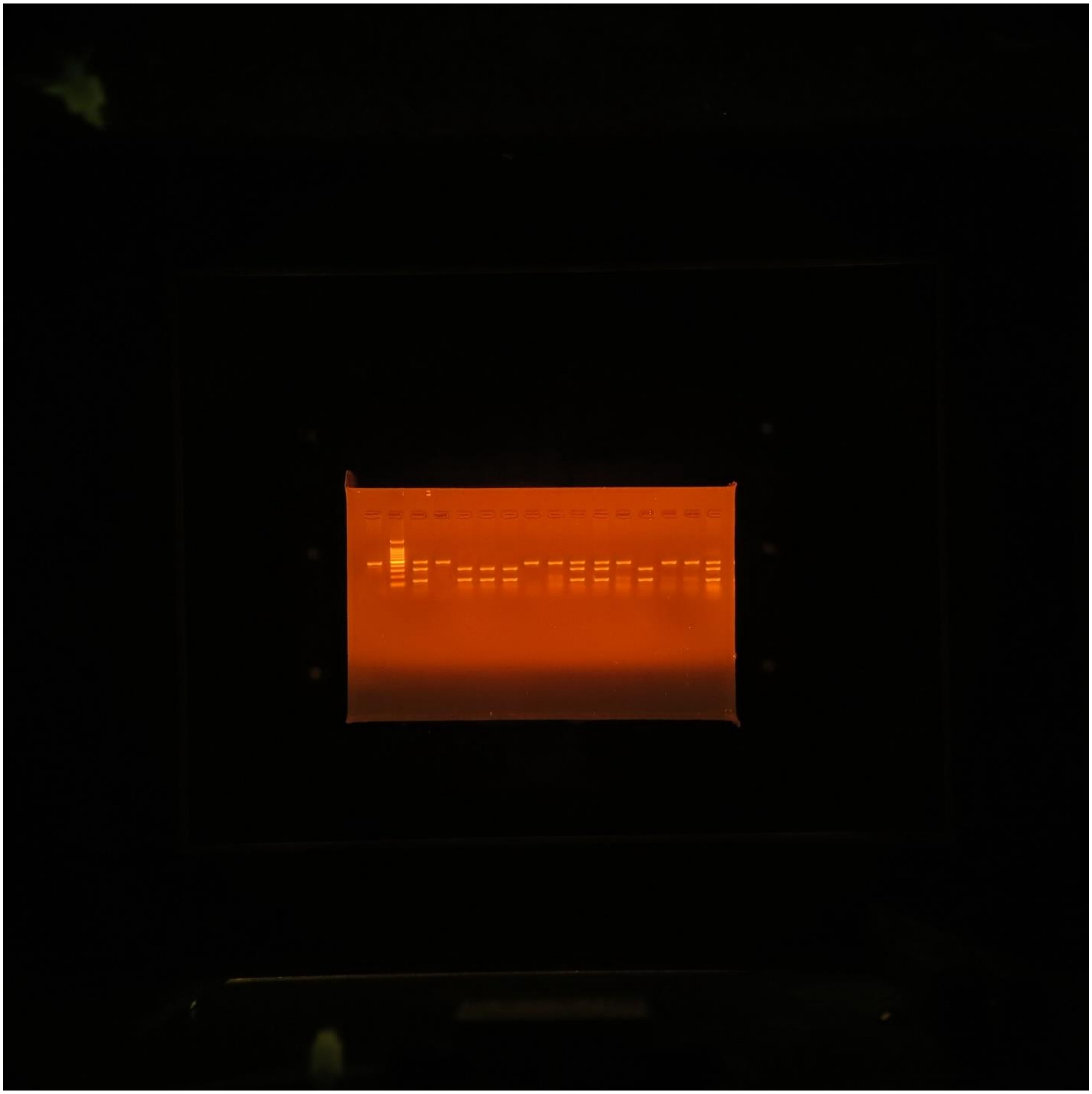

Adipo+45T-G PCR product

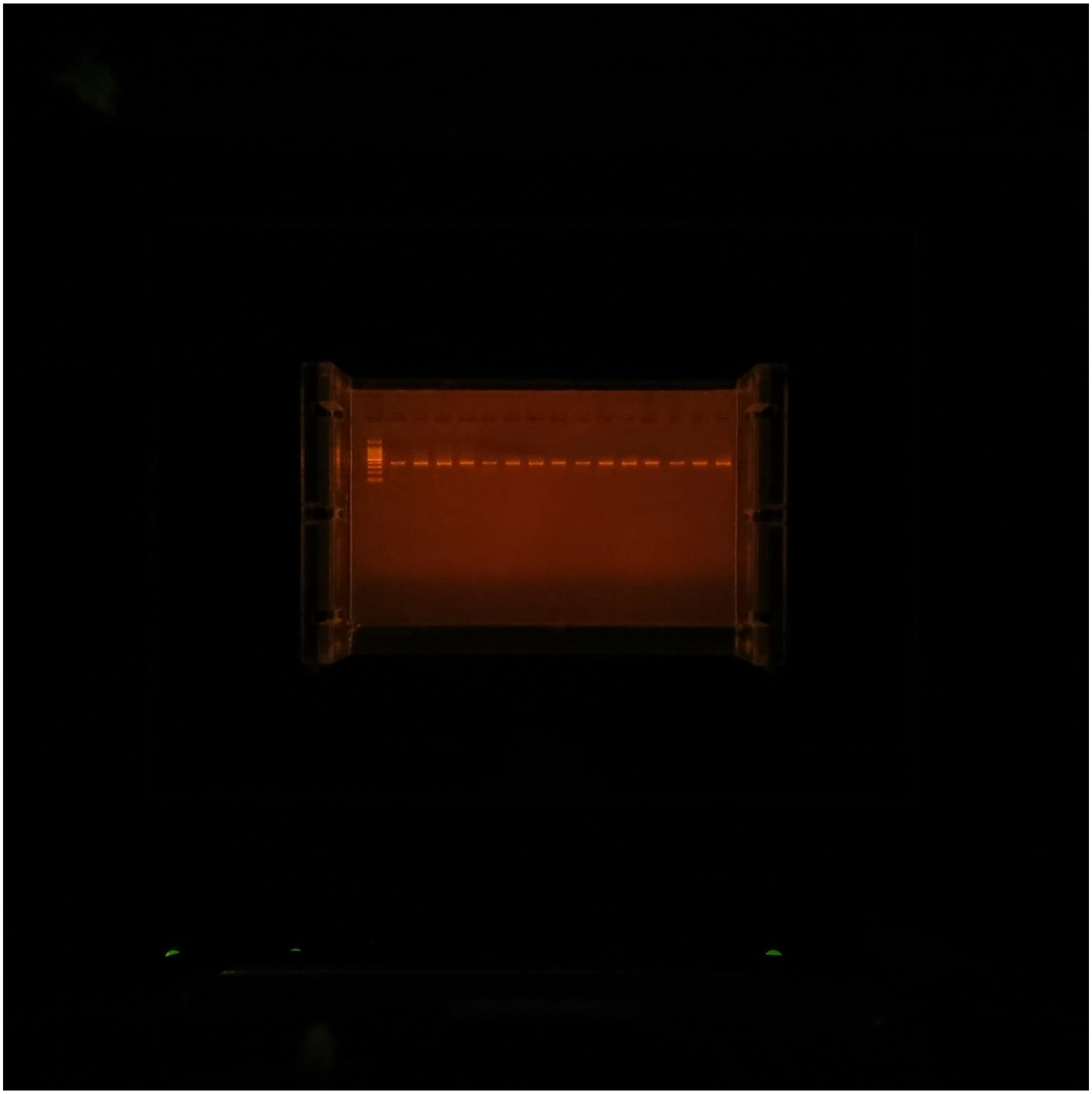

Adipo-11391 PCR after digestion

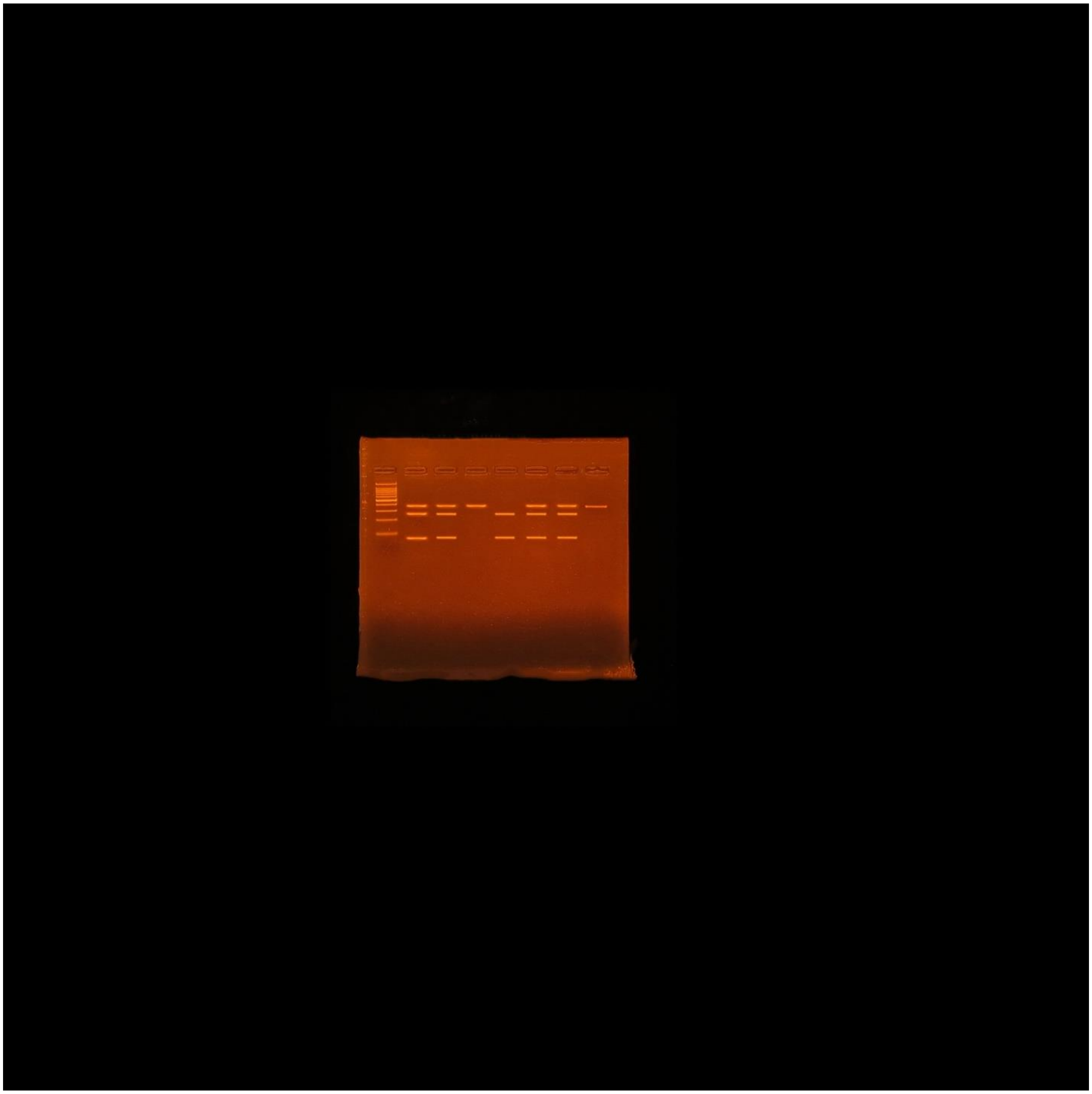

Supplement: Supplementary file 1 — Supplementary Material 1 [file 41598_2025_86143_MOESM1_ESM.pdf]
